# Association between vitamin D deficiency and hypothyroidism: results from the National Health and Nutrition Examination Survey (NHANES) 2007–2012

**DOI:** 10.1186/s12902-021-00897-1

**Published:** 2021-11-12

**Authors:** Sandeep Appunni, Muni Rubens, Venkataraghavan Ramamoorthy, Anshul Saxena, Raees Tonse, Emir Veledar, Peter McGranaghan

**Affiliations:** 1 Department of Biochemistry, Government Medical College, Kozhikode, Kerala India; 2 Miami Cancer Institute, Miami, Florida USA; 3 Baptist Health South Florida, Miami, Florida USA; 4 Department of Biostatistics, Florida International University, Miami, FL USA; 5 Division of Cardiology, Emory University School of Medicine, Atlanta, GA USA; 6 Department of Internal Medicine and Cardiology, Charité - Campus Virchow-Klinikum, Augustenburger Platz 1, 13353 Berlin, Germany

**Keywords:** Hypothyroidism, Low vitamin D, Autoimmune, Thyroid peroxidase, Thyroglobulin

## Abstract

**Purpose:**

Many smaller studies have previously shown a significant association between thyroid autoantibody induced hypothyroidism and lower serum vitamin D levels. However, these finding have not been confirmed by large-scale studies. In this study, we evaluated the relationship between hypothyroidism and vitamin D levels using a large population-based data.

**Methods:**

For this study, we used National Health and Nutrition Examination Survey (NHANES) during the years 2007–2012. We categorized participants into three clinically relevant categories based on vitamin D levels: optimal, intermediate and deficient. Participants were also split into hypothyroid and hyperthyroid. Weighted multivariable logistic regression analyses were used to calculate the odds of being hypothyroid based on vitamin D status.

**Results:**

A total of 7943 participants were included in this study, of which 614 (7.7%) were having hypothyroidism. Nearly 25.6% of hypothyroid patients had vitamin D deficiency, compared to 20.6% among normal controls. Adjusted logistic regression analyses showed that the odds of developing hypothyroidism were significantly higher among patients with intermediate (adjusted odds ratio [aOR], 1.7, 95% CI: 1.5–1.8) and deficient levels of vitamin D (aOR, 1.6, 95% CI: 1.4–1.9).

**Conclusion:**

Low vitamin D levels are associated with autoimmune hypothyroidism. Healthcare initiatives such as mass vitamin D deficiency screening among at-risk population could significantly decrease the risk for hypothyroidism in the long-term.

## Introduction

Hypothyroidism is an endocrine disorder characterized by lower serum levels of thyroxin, resulting in clinical spectrum that varies from no signs and symptom to life threatening complications [1, 2]. The major circulating thyroid hormone consists of thyroxine (Total T4), triiodothyronine (Total T3) and their free forms, free T4 (fT4) and free T3 (fT3) [3]. Blood levels are tightly regulated by the hypothalamus-pituitary-thyroid axis through a negative feedback mechanism [4].^.^ Classically, primary overt hypothyroid cases will have low serum levels of fT4 and reciprocally elevated thyroid stimulating hormone (TSH) [1, 5]. The disease spectrum also consists of an occult condition known as subclinical hypothyroidism which is characterized by normal total T4 levels but modestly elevated TSH [6]. As per National Health and Nutrition Examination Survey (NHANES III) of United Sates population (1988 to 1994) the overall prevalence of hypothyroidism is 4.6% of which 0.3% have overt and 4.3% have subclinical hypothyroidism [6]. Hypothyroidism due to iodine deficiency is highly prevalent in geographical regions where the soil is deficient in iodine, such as hilly and mountainous terrain, whereas in iodine sufficient areas autoimmune etiology (Hashimoto’s thyroiditis) predominates [7].

Vitamin D is a fat-soluble nutrient that is canonically converted in vivo to active hormone (calcitriol or 1,25-dihydroxycholecalciferol) following two hydroxylation steps, first in the liver (calcidiol or 25-hydroxy vitamin D), and second in the kidneys. Circulating vitamin D status is evaluated by quantitation of serum 25-hydroxy vitamin D. Vitamin D has two forms. Namely vitamin D_2_ and vitamin D_3_. Vitamin D_2_ is obtained from plant sterol ergosterol and vitamin D_3_ (cholecalciferol) is derived from cholesterol under the skin [8]. Vitamin D is essential for maintenance of healthy body systems including the immune system and has protective role in cancer prevention [9]. Vitamin D deficiency is prevalent in both developed and developing countries and is determined by low serum 25-hydroxy vitamin D (< 25 nmol/l) levels [10]. In the US, overall vitamin D deficiency prevalence rate was 41.6% and highest in blacks followed by Hispanics [11].

Hashimoto’s thyroiditis is characterized by hemopoietic lymphocytic infiltration and subsequent autoimmune mediated destruction of the thyroid follicles resulting in variable clinical presentations, ranging from euthyroid to subclinical to frank hypothyroid state with or without evident goiter [12]. Different clinicopathological types have been reported and is characterized by circulating antibodies against thyroid peroxidase (TPO) and thyroglobulin (Tg). TPO is primarily involved in the synthesis of thyroid hormone (T4 and T3) while Tg sequesters thyroid hormones within thyroid follicles [12, 13]. Epidemiologically, the incidence is more common in females than in males and lower among alcoholics and smokers [14]. Etiopathogenesis involves genetic predisposition and environmental factor that are newly associated with vitamin D and selenium deficiency. Vitamin D has immunoregulatory and anti-inflammatory functions such as regulating the activity of the adaptive immune system, especially the low dendritic cell differentiation, enhanced Th2 helper cells (shifting from Th1 to Th2 helper cells) maturation, and activation of T regulatory (Treg) cells [15]. Low vitamin D levels are significantly correlated with the development of autoimmune diseases such as rheumatoid arthritis, systemic lupus erythematosus, systemic sclerosis, type 1 diabetes mellitus, multiple sclerosis, and autoimmune thyroid diseases [16]. A meta-analysis that looked for vitamin D levels in autoimmune thyroiditis showed that both Hashimoto thyroiditis and Grave’s disease were associated with lower vitamin D levels [17]. Conversely, some studies have shown that there was no significant association between vitamin D and auto immune thyroiditis [18]. In this study, we observed the relationship between vitamin D and hypothyroidism using a large nationally representative NHANES data.

## Methods and materials

### Study design

For this study, we used National Health and Nutrition Examination Survey (NHANES) during the years 2007–2012. The NHANES is a nationwide survey on the health and nutritional status of the US population and include all noninstitutionalized people and is conducted by the National Center for Health Statistics (NCHS), Centers for Disease Control and Prevention (CDC). NHANES uses complex, multistage cluster probability sampling design for conducting the survey and participants are randomly selected for the survey. Data for the survey are gathered by conducting interviews of the participants at their homes, and a selected number of these participants are invited for medical examinations and laboratory evaluations done at Mobile Examination Centers (MECs). Survey protocol for NHANES data collection is approved by the NCHS institutional review board.

### Study population

For this study, we used data from participants ≥20 years of age. All NHANES participants who were ≥ 18 years of age had signed a written informed consent following an extensive and detailed description about the survey including the interview, medical examination and laboratory evaluation. Details of the of the methods and protocols for the questionnaires, laboratory, and examination can be found elsewhere [19].

### Vitamin D status

For this study, we categorized participants into three clinically relevant categories based on the serum 25(OH) D levels following the Endocrinology Society Clinical Practice Guidelines [20]. The three categories are optimal (≥30 ng/mL), intermediate (20 to < 30 ng/mL) and deficient (< 20 ng/mL) vitamin D levels.

### Hypothyroidism

We used the laboratory reference range of thyroid stimulating hormone (TSH), 0.34–5.60 mIU/L, from manufacturer’s studies, for diagnosing hypothyroidism [21]. Participants were defined as hypothyroid if their TSH was more than 5.60 mIU/L or were on levothyroxine. Participants were categorized as normal controls if their TSH was between 0.34–5.60 mIU/L and they were not taking any thyroid medication.

The study was reviewed by the Miami Cancer Institute’s Institutional Review Board, which exempted the study from institutional review board approval and waived the requirement for informed consent because it uses previously collected deidentified data stored in NHANES.

### Statistical analysis

Statistical analysis was performed using SAS (version 9.4, SAS Institute, Cary, North Carolina), which accounted for the complex survey design and clustering. Demographic and socioeconomic measures were compared between hypothyroid patients and normal controls using independent samples t test for continuous variables and Chi-square test for categorical variables. Similarly, clinical characteristics of hypothyroid patients and normal controls were compared. Weighted multivariable logistic regression analyses were used to calculate the odds of being hypothyroid based on vitamin D status, after adjusting for covariates such as age, education, income, smoking, alcohol consumption, body mass index (BMI), physical activity, hypertension, diabetes, dyslipidemia, blood urea nitrogen, creatinine and magnesium levels. The NHANES measures physical activity using the physical activity questionnaire. Whether the recommended level of physical activity was met (yes or no) was ascertained through this questionnaire, which included the duration of moderate to vigorous intensity leisure-time physical activities performed for a minimum of 10 min at a time in the previous month. Age, race, education, income, smoking, alcohol consumption, and physical activity were self-reported by participants during house visits for surveys. Height and weight for BMI, systolic and diastolic blood pressures, fasting blood glucose, glycated haemoglobin, serum lipid profile, blood urea nitrogen, creatinine and magnesium levels were measured by trained healthcare professionals during Medical Examination Center visits. Since the proportion of missing data was small and not missing completely on random, NOMCAR option was used during the regression analysis. Statistical significance was set at *P* < 0.05.

## Results

A total of 7943 participants were included in this study, of which 614 (7.7%) were having hypothyroidism. The man age of this cohort was 47.0 (SE = 0.3) years and 51.5% were females. Majority of the participants were white (69.7%), followed by blacks (10.2%) and Mexican Americans (8.1%). About 57.5% of the participants had more than 12 years of formal education. About 37.4% of participants were in the highest income group and 14.3% were in the lowest income group. Majority of the participants (80.8%) reported that they had health insurance. More than half of the participants (58.1%) reported that they had engaged in some form of physical activity and 75.8% never smoked and 78.1% currently consumed alcohol. Significant differences were observed in all demographics and socioeconomic factors between hypothyroid patients and normal control (Table 1).

**Table 1:** Demographic and socioeconomic characteristics of the participants, NHANES 2007–2012 (*n* = 7943)

Variables	Hypothyroidism ***N*** = 614 (7.7%)	Normal Control ***N*** = 7329 (92.3%)	***P*** value	Total ***N*** = 7943
Age, mean years (SE)	51.2 (0.8)	46. 1 (0.3)	< 0.001	47.0 (0.3)
Female, % (SE)	76.0% (2.4)	49.4% (0.5)	< 0.001	51.5% (0.5)
Race/ethnicity, % (SE)			< 0.001	
Non-Hispanic White	86.8% (2.0)	68.2% (2.4)		69.7% (2.3)
Non-Hispanic Black	3.3% (0.6)	10.8% (1.2)		10.2% (1.1)
Mexican American	4.1% (0.9)	8.5% (1.1)		8.1% (1.0)
Other race	5.8% (1.3)	12.5% (1.2)		12% (1.2)
Education, % (SE)			0.047	
Less than 12	15.2% (2.0)	19.4% (1.1)		19% (1.0)
12	22.9% (2.0)	23.5% (0.8)		23.5% (0.8)
More than 12	62.0% (3.0)	57.1% (1.5)		57.5% (1.5)
FPIR, % (SE)			< 0.001	
< 1 (Lowest income)	7.9% (1.4)	14.9% (0.9)		14.3% (0.9)
1 to < 2	21.6% (1.7)	20.7% (0.9)		20.7% (0.9)
2 to < 4	31.7% (2.3)	27.2% (1.0)		27.5% (1.0)
≥4 (Highest income)	38.7% (3.0)	37.3% (1.5)		37.4% (1.5)
Health insurance, % (SE)	92.6% (1.1)	79.8% (0.8)	< 0.001	80.8% (0.8)
Physical activity, % (SE)	47.5% (3.7)	59% (0.8)	0.001	58.1% (0.9)
Smoking, % (SE)			0.002	
Current	13.3% (1.7)	21.8% (0.9)		21.1% (0.8)
Former	2.9% (1.1)	2.9% (0.3)		2.9% (0.3)
Never	83.6% (1.8)	75.1% (0.9)		75.8% (0.8)
Alcohol use, % (SE)				
Never	13.9% (1.8)	9.8% (0.6)	0.010	10.1% (0.5)
Former	12% (1.3)	11.7% (0.6)		11.8% (0.5)
Current	74.1% (2.1)	78.5% (0.8)		78.1% (0.8)

**Fig. 1:**
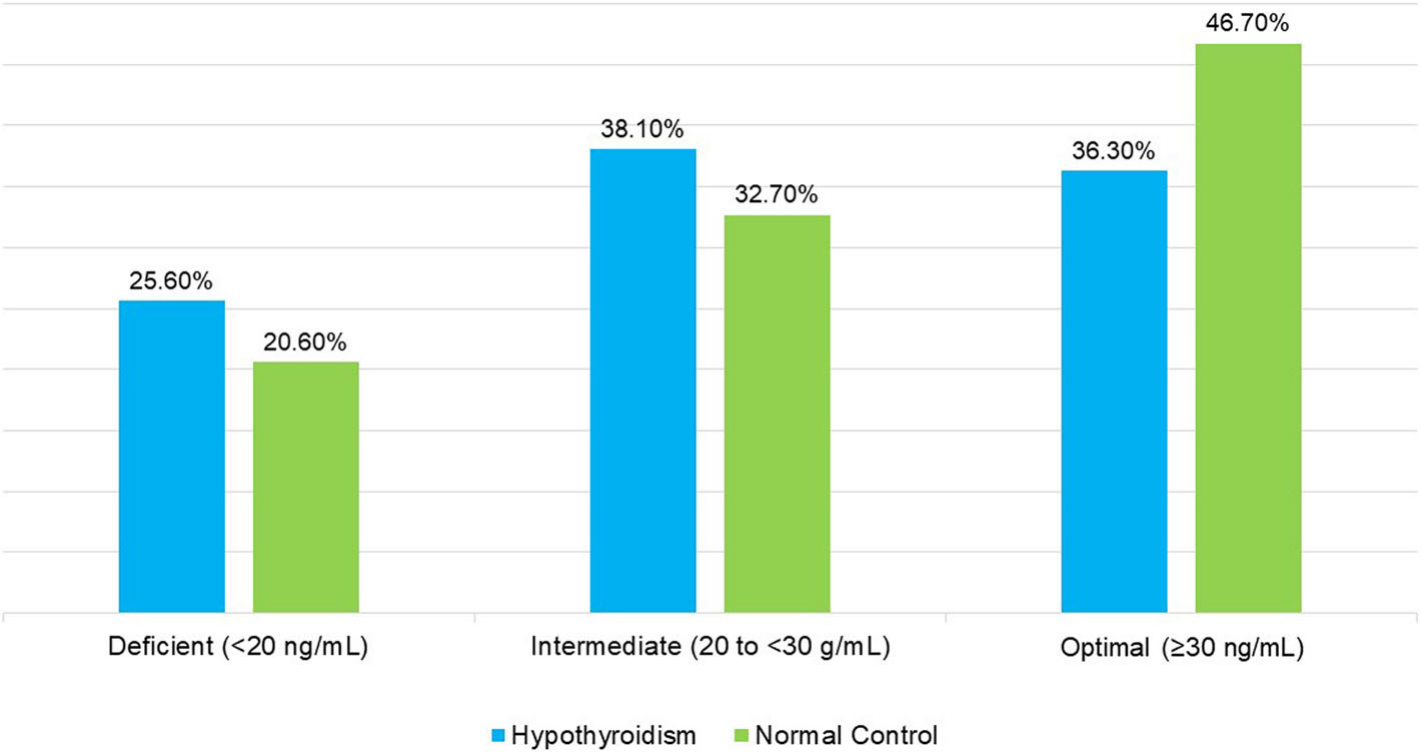
Comparison of vitamin D categories by hypothyroidism and normal control

Comparison of clinical characteristics between hypothyroid patients and normal controls also showed significant difference in majority of the factors. There was a significant association (*P* < 0.001) between vitamin D categories and hypothyroid state (Fig. 1). Nearly 25.6% of hypothyroid patients had vitamin D deficiency, compared to 20.6% in normal controls. Majority of participants in the hypothyroid group were obese (40.5%), while 33.4% were obese in the normal controls. Significantly higher proportion of participants in the hypothyroid group were hypertensive (47.1% versus 29.5%, *P* < 0.001), diabetic (19.7% versus 10.7%, *P* < 0.001), and dyslipidemic (54.9% versus 44.7%, *P* < 0.001). Similarly, there were significant mean differences in majority of biochemical variables between hypothyroid patients and normal controls. Details are shown in Table 2.

**Table 2:** Clinical characteristics of the participants, NHANES 2007–2012 (N = 7943)

Variables	Hypothyroidism ***N*** = 614	Normal Control ***N*** = 7329	***P*** value	Total ***N*** = 7943
Vitamin D categories, % (SE)			< 0.001	
Deficient (< 20 ng/mL)	25.6% (1.9)	20.6% (1.3)		21.0% (1.2)
Intermediate (20 to < 30 g/mL)	38.1% (1.9)	32.7% (0.8)		33.1% (0.7)
Optimal (≥30 ng/mL)	36.3% (1.7)	46.7% (1.2)		45.9% (1.1)
BMI (kg/m^2^), % (SE)			< 0.001	
Underweight (< 18.5 kg/m^2^)	1.3% (0.6)	1.8% (0.2)		1.7% (0.2)
Normal (18.5–24.9 kg/m^2^)	25.3% (1.6)	30.2% (0.8)		29.8% (0.7)
Overweight (25.0–29.9 kg/m^2^)	33.0% (2.8)	34.2% (0.8)		34.1% (0.8)
Obese (≥30.0 kg/m^2^)	40.5% (3.0)	33.8% (0.8)		34.4% (0.8)
Hypertension, % (SE)	47.1% (2.9)	29.5% (0.7)	< 0.001	30.9% (0.7)
Diabetes, % (SE)	19.7% (2.4)	10.7% (0.5)	< 0.001	11.5% (0.5)
Dyslipidemia, % (SE)	54.9% (2.3)	44.7% (0.8)	< 0.001	45.5% (0.7)
Thyroglobulin antibodies (IU/mL)	68.1 (3.2)	6.4 (0.8)	< 0.001	11.3 (1.3)
Thyroid peroxidase antibodies (IU/mL)	87.2 (8.6)	15.8 (1.2)	< 0.001	21.3 (1.2)
Total protein (g/L)	70.7 (0.3)	71.3 (0.1)	0.145	71.2 (0.1)
Albumin (g/L)	41.9 (0.2)	42.9 (0.1)	< 0.001	42.8 (0.1)
Alanine aminotransferase (U/L)	25.2 (1.1)	25.9 (0.2)	0.522	25.8 (0.2)
Aspartate aminotransferase (U/L)	27.6 (1.2)	25.9 (0.2)	0.211	26 (0.2)
Alkaline phosphatase (U/L)	68.6 (1.4)	66.7 (0.4)	0.139	66.8 (0.4)
Total bilirubin (mg/dL)	0.74 (0.1)	0.8 (0.1)	0.068	0.8 (0.1)
Iodine, urine (ug/L)	381.7 (63.2)	248.8 (12.6)	0.077	259.5 (13.6)
Direct HDL-Cholesterol (mg/dL)	55 (1.1)	52.2 (0.3)	0.008	52.4 (0.3)
LDL-cholesterol (mg/dL)	117 (1.0)	116.4 (0.6)	0.849	116.4 (0.6)
Total Cholesterol (mg/dL)	198.3 (1.2)	196.9 (0.7)	0.514	197 (0.6)
Triglyceride (mg/dL)	131.8 (2.2)	134 (2.9)	0.710	133.8 (2.6)
Creatinine (mg/dL)	0.88 (0.1)	0.9 (0.01)	0.434	0.9 (0)
Blood urea nitrogen (mg/dL)	14.4 (0.2)	12.9 (0.1)	< 0.001	13 (0.1)
Chloride (mmol/L)	103.3 (0.2)	104 (0.1)	< 0.001	104 (0.1)
Phosphorus (mg/dL)	3.8 (0.1)	3.8 (0.1)	0.002	3.8 (0.1)
Potassium (mmol/L)	4.0 (0.1)	4.0 (0.1)	0.059	4.0 (0.1)
Sodium (mmol/L)	139 (0.1)	139.2 (0.1)	0.226	139.2 (0.1)
Total calcium (mg/dL)	9.4 (0.1)	9.4 (0.2)	0.018	9.4 (0.2)

Unadjusted logistic regression analyses or those adjusted for selected covariates (age, sex, race, smoking, alcohol consumption, BMI and physical activity) did not show any association between vitamin D deficiency and hypothyroidism. However, logistic regression analyses adjusted for age, race, education, income, smoking, alcohol consumption, BMI, physical activity, hypertension, diabetes, dyslipidemia, blood urea nitrogen, creatinine and magnesium showed that the odds of developing hypothyroidism were significantly higher among patients with intermediate (adjusted odds ratio [aOR], 1.7, 95% CI: 1.5–1.8) and deficient levels of vitamin D (aOR, 1.6, 95% CI: 1.4–1.9). All regression models are shown in Table 3. Table 4 shows correlation between variables included in the regression models.

**Table 3:** Multivariable logistic regression results showing association between vitamin D and hypothyroidism (*N* = 7943)

Models	Optimal (≥30 ng/mL)	Intermediate (20 to < 30 g/mL)	Deficient (< 20 ng/mL)
Model 1	Reference	1.4 (0.7–1.6)	1.1 (0.7–1.4)
Model 2	Reference	1.2 (0.7–1.4)	1.3 (0.6–1.7)
Model 3	Reference	1.2 (0.5–1.4)	1.2 (0.4–1.6)
Model 4	Reference	1.3 (0.8–1.5)	1.2 (0.6–1.7)
Model 5	Reference	1.2 (0.7–1.4)	1.3 (0.3–1.7)
Model 6	Reference	1.6 (0.9–1.6)	1.5 (0.3–1.8)
Model 7	Reference	**1.7 (1.5–1.8)**	**1.6 (1.4–1.9)**

Model 1: Unadjusted

Model 2: Adjusted for age, sex, education, and income

Model 3: Adjusted for age, sex, education, income, smoking, alcohol consumption, BMI and physical activity

Model 4: Adjusted for age, education, income, smoking, alcohol consumption, BMI, physical activity, hypertension, and diabetes

Model 5: Adjusted for age, education, income, smoking, alcohol consumption, BMI, physical activity, hypertension, diabetes, dyslipidemia, and blood urea nitrogen

Model 6: Adjusted for age, education, income, smoking, alcohol consumption, BMI, physical activity, hypertension, diabetes, dyslipidemia, blood urea nitrogen, and creatinine

Model 7: Adjusted for age, education, income, smoking, alcohol consumption, BMI, physical activity, hypertension, diabetes, dyslipidemia, blood urea nitrogen, creatinine, and magnesium

**Table 4:** Correlation between variables included in the regression models

	1	2	3	4	5	6	7	8	9	10	11	12	13	14	15
1. Age	–														
2. Education	0.152	–													
3. Income	0.250	0.243	–												
4. Smoking	0.187	0.015	0.163	–											
5. Alcohol consumption	0.224	0.197	0.159	0.229	–										
6. Body mass index	0.250	0.200	0.123	0.462	0.476	–									
7. Physical activity	0.552	0.063	0.116	0.044	0.167	0.352	–								
8. Hypertension	0.091	0.293	0.254	0.368	0.018	0.478	0.284	–							
9. Diabetes	0.187	0.187	0.247	0.260	0.169	0.167	0.173	0.142	–						
10. Dyslipidemia	0.398	0.232	0.317	0.457	0.145	0.196	0.084	0.140	0.122	–					
11. Blood urea nitrogen	0.407	0.180	0.318	0.343	0.354	0.168	0.374	0.213	0.184	0.243	–				
12. Creatinine	0.351	0.246	0.169	0.107	0.342	0.182	0.486	0.752	0.388	0.105	0.203	–			
13. Magnesium	0.080	0.309	0.451	0.188	0.415	0.106	0.239	0.093	0.364	0.114	0.327	0.117	–		
14. Vitamin D	0.166	0.425	0.179	0.302	0.341	0.040	0.327	0.143	0.499	0.440	0.305	0.302	0.341	–	
15. Hypothyroidism	0.370	0.128	0.167	0.135	0.167	0.098	0.156	0.228	0.167	0.222	0.470	0.344	0.086	0.355	–

## Discussion

This study evaluated the association between vitamin D levels and hypothyroidism using a large nationally representative database. Earlier studies on smaller populations have shown an inverse relationship between vitamin D levels and the occurrence of hypothyroidism [22–25]. We have studied the association in 7943 participants who enrolled in the NHANES during the period 2007–2012 and included 614 hypothyroid participants.

In our study, hypothyroid group had a mean age significantly higher than the control participants and majority of participant cases were females. Similar findings were found in a study by Mackawy et al^.^ which had greater number of female subjects with hypothyroidism [25]. Other studies done in middle income countries such as India as reported by Velayutham et al. and Unnikrishnan et al. also had greater number of female patients with hypothyroidism [26, 27]. This shows that regular thyroid evaluation of females early in the middle ages is necessary to diagnose and initiate treatment in the early course of the illness. Kim et al. observed that premenopausal females are at a higher risk of developing autoimmune hypothyroidism compared to men and even postmenopausal females [28].

Majority of the hypothyroid subjects in our study were non-Hispanic whites. Schectman et al. studied 809 age and sex matched suspected cases of hypothyroidism and observed that mean TSH in blacks were significantly lower compared to whites [29]. This study inferred that racial parameter contributed to 6.5% variation in TSH levels. Olmos et al. in the ELSA-Brazil (Brazilian longitudinal study on adult health) study observed higher prevalence of overt hypothyroidism in whites, compared to brown and black population [30]. It signifies that brown and black ethnicity may have a protective effect from developing overt hypothyroidism. We also observed that a significant number of participants belonged to the highest income strata and majority had good education. This suggests that hypothyroid participants have the potential to comprehend the impact of the illness and take the necessary steps for effective treatment of the ailment. Similar findings were observed by Olmos et al. in their Brazilian population, where most of the hypothyroid subjects taking levothyroxine belonged to the high socioeconomic strata [30]. About 92% of hypothyroid participants in our study had health insurance coverages which could cover the medication cost.

In our study, lifestyle characteristics of participants showed that they had lower levels of physical activity and consumed alcohol, though majority of them never smoked. Ciloglu et al. reported that increased physical activity at the anaerobic threshold steadily increased the production of TSH, fT4, and T4 but produced a fall in total T3 and fT3 [31]. Thus, regular aerobic exercises may be beneficial in normal subjects to boost endogenous thyroid hormone synthesis. Bansal et al. reported that regular exercise could have a beneficial effect on the thyroid hormone status of hypothyroid subjects on treatment [32]. In our study, a significant majority of hypothyroid participants consumed alcohol. It is essential to note that alcohol has a negative effect over the thyroid function. Alcohol reduces the levels of peripheral thyroid hormones especially during late alcohol withdrawal and can aggravate hypothyroidism [33, 34]. Thus, abstinence from alcohol should be advised for patients diagnosed with hypothyroidism and who are on thyroid medications.

Vitamin D levels could be influenced by whether the physical activity is performed indoors or outdoors. Similarly, it could also be influenced by alcohol consumptions, which significantly increases vitamin D levels. Although, participants in our study reported lower levels of physical activity and alcohol consumption, there were no interaction of these variables with the relationship between vitamin D status and hypothyroidism.

Vitamin D deficiency is being recognized as a global pandemic most presumably due to heliophobia of the general population, especially among those residing in southeast Asia [35]. Thus, endocrinologist in such countries have recommended to increase the recommended daily allowance (RDA) for vitamin D to prevent deficiency states. Huotari and Herzig in their literature review on vitamin D status in populations living in higher latitudes recommended that vitamin D deficiency was highly predominant in the winter season and consumption of vitamin D fortified foods and supplements would be necessary to prevent deficiency states in these seasons [36]. Parva et al. using NHANES found that the prevalence of vitamin D deficiency was as high as 39% in the US population [37]. They also found out that the odds of having vitamin D deficiency were higher among black race and individuals with poor health and obesity. One of the key clinical condition observed in vitamin D deficiency was the increased incidence of autoimmune diseases [16]. Hypothyroidism is commonly a disease resulting from similar autoimmune insults as indicated by higher anti-peroxidase and anti-thyroglobulin antibodies [12, 13].

We found that participants having deficient vitamin D levels were significantly higher among hypothyroid participants, compared to normal controls. Conversely, participants with optimal vitamin D levels were significantly higher in control group, compared to hypothyroid participants. In a nutshell, 63.7% of the hypothyroid participants has sub-optimal levels of vitamin D as compared to controls (53.3%). This shows that prevalence of low vitamin D levels could be associated with increased risk of developing hypothyroidism, though we could not establish a causal relationship. Mackawy et al. in a small sample size of 30 subjects observed that vitamin D deficiency is associated with lower thyroid levels [25].

In our study, we found that the odds of developing hypothyroidism were significantly higher among patients with intermediate and deficient levels of vitamin D. Kim^38^ reported in Korean population that vitamin D deficiency is highly prevalent in autoimmune Hashimoto’s thyroiditis presenting with overt hypothyroidism than subclinical hypothyroid variants [38]. Studies have shown that a reciprocal relationship exists between serum TSH and vitamin D levels in hypothyroid subjects [38, 39]. ElRawi et al. reported vitamin D deficient hypothyroid subjects have higher insulin resistance which significantly correlated with higher anti-thyroid antibodies, anti-TPO and anti-Tg [39]. However, they did not observe any significant vitamin D receptor polymorphism in subject vs control suggesting that correction with vitamin D supplements may possibly have a therapeutic benefit in correcting thyroid status. Talaei et al. found that supplementation of 50,000 IU vitamin D to hypothyroid subject lowered TSH and parathormone levels without any significant effect over serum thyroxine (T3 and T4) levels [40]. In another scenario, Ucan et al. observed that a similar vitamin D oral supplementation to autoimmune Hashimoto’s thyroiditis subjects significantly improved thyroid status concurrent with a decrease in autoimmune antibodies and an increase in free T4 (fT4) levels [41]. Mirhosseini et al. in a large cohort found that a significant fraction of subjects had an improvement in their thyroid status following vitamin D supplementation [42]. A randomized control trial by Chahardoli et al. found that vitamin D supplementation significantly reduced the levels of tropic hormone TSH and anti-Tg antibodies [43]. However, this study did not find any significant differences with respect to anti-TPO levels and thyroxine levels between the groups. These finding suggest that vitamin D has a key role in regulating both the thyroid destroying autoimmune antibodies as well as the pituitary trophic hormone TSH. In another study, thyroidectomy in the past as well as subjects receiving thyroid supplementation therapy had higher vitamin D levels, compared to undertreated patients [44]. This suggest that undertreated hypothyroidism and progressive autoimmune inflammation mediated destruction of thyroid could have detrimental effect over vitamin D metabolism and could potentiate the systemic ill-health effects associated with thyroid dysfunction. Meta-analysis and case control study-based observations show that individuals with low vitamin D levels also have an increased risk of developing thyroid cancer [45].

In our study, we found a significant association between body mass index and hypothyroid state. Obesity or increased fat deposition is another risk factor for low vitamin D levels. Steroid derived hormones like vitamin D tend to get redistributed in the adipose tissue which function as a huge reservoir [46]. This results in a lower plasma level of circulating pre-vitamin D resulting in vitamin D deficiencies. Liel et al. reported low levels of circulating vitamin D in obese subjects compared to non-obese [47]. Vitamin D deficient hypothyroid patients also show an increased likelihood for developing diabetes mellitus, hypertension and anemia [48]. McGill et al. reported that vitamin D levels fall by 0.29 nmol/L for every centimeter increase in waist circumference due to abdominal obesity [49]. Hypothyroidism due to autoimmune destruction is highly associated with centripetal obesity with metabolic derangements where both anti-TPO and anti-Tg positively correlate with serum triglyceride levels and waist circumference [50]. Our study also show that hypothyroid subjects had significantly increased prevalence of diabetes mellitus, hypertension and dyslipidemia thus qualifying for metabolic syndrome. This suggest that metabolic alterations associated with hypothyroidism could be plausibly aggravated by vitamin D deficiency.

We used NHANES, which is a nationally representative data set. Because of the large sample size, we could make better estimates of the relationship between vitamin D and hypothyroidism. Therefore, our results would be generalizable to the entire U.S. population thereby assuring high levels of external validity. Most of the variables in our study are based on laboratory results. Hence, we could assure greater levels of internal validity of our results. Our study has some limitations. Since this study is cross-sectional, we could not infer causality in the association between vitamin D and hypothyroidism. In addition, we also excluded missing data which could have led to some confounding in the results. Due to the limitations NHANES data certain important variables related to vitamin D such as indoor versus outdoor physical activity, seasonal changes, and geographical coordinates could not be ascertained and could have led to some biases. Pathological conditions such as non-alcoholic fatty liver disease which could affect vitamin D also could not be determined. Some of the variables collected in the study such as smoking status, alcohol consumption, and physical activity, were self-reported and therefore susceptible to social desirability bias. Though we tried our best to account for potential confounders and covariates some residual confounders could still affect the findings in our study.

## Conclusion

Our study highlights the association between vitamin D deficiency and hypothyroidism using a large nationally representative data. Future largescale experimental studies are needed to confirm the findings in our study. Based on the findings of our study, healthcare initiatives such as mass vitamin D deficiency screening among at-risk population such as elderly, obese, indoor and sedentary individuals and prompt treatment with dietary supplementations could significantly decrease the risk for hypothyroidism in the long-term.

## Data Availability

The dataset used in this study is publicly available from https://www.cdc.gov/nchs/nhanes/index.htm
